# Deep vein thrombosis after insertion of a central venous catheter: a case report

**DOI:** 10.11604/pamj.2022.42.25.34452

**Published:** 2022-05-11

**Authors:** Ihdinal Mukti, Artaria Tjempakasari

**Affiliations:** 1 Department of Internal Medicine, Faculty of Medicine, Airlangga University, Dr. Soetomo Hospital, Surabaya, Indonesia,; 2 Nephrology Division, Department of Internal Medicine, Faculty of Medicine, Airlangga University, Dr. Soetomo Hospital, Surabaya, Indonesia

**Keywords:** Deep vein thrombosis, hemodialysis, double lumen catheter, case report

## Abstract

A male patient aged 50 years, presented with a swelling in the right leg which had occurred five days before hospital admission. This was associated with pain in the swollen leg. The patient had a history of femoral double lumen catheter (DLC) insertion for hemodialysis. On physical examination, Wong Baker's scale was 3, Wells' score was 3, and the patient had edematous, red, and warm right lower extremity. Laboratory results showed anemia (Hb 11.4 g/dl), leukocytosis (27.99 x 10^3^/ul), increased blood urea nitrogen (BUN) (60 mg/dl), increased serum creatinine (9.93 mg/dl), hyperkalemia (6 mmol/l), urine leukocytes +3; 15-20/hpf. Lower extremity ultrasound revealed diffuse deep vein thrombosis (DVT). After the diagnosis of DVT, the patient was treated with fondaparinux injection and warfarin as an anticoagulant for six days. Our results show that treatment was effective in reducing swelling and pain in the right leg, that disappeared after a three months´ treatment.

## Introduction

Double lumen catheters, for semi-permanent or temporary access, have been widely used in patients on hemodialysis. This access is needed for the exchange transfusion between the patient and the dialysis machine during the hemodialysis process. Double lumen catheters are widely chosen and used by outpatient hemodialysis patients because of their ease of use in dialysis procedures [1]. Double lumen catheter also has other advantages. It is more comfortable for patients since it is painless and can be used repeatedly [2]. Although double lumen catheter is considered safe and has various advantages, it can lead to complications. Since it was first used, several complications during the installation phase have been reported. They include local hematoma, infection, thrombosis, arterial perforation, arteriovenous fistula, pseudo aneurysm and even air embolism [3]. The incidence of severe complications due to double-lumen catheter insertion ranges from 0.4% to 9.9% [4]. A cross-sectional research of complications due to double lumen catheter insertion showed that 28 out of 200 patients had complications. Of the 28 patients who had complications, 28.57% had arterial perforation, 14.28% had kinking/mal-positioning, and 7.14% had arteriovenous fistulas. Another research showed outcomes among 161 patients, 22 of whom had thrombosis (16.7%) due to catheter malfunction, and 29 patients (22.0%) had infection due to catheter insertion [5].

Thrombosis can occur after catheter insertion, with an average incidence of 14-18% [6]. Thrombosis is more common during femoral venous catheter insertion than during the placement of catheter in the internal jugular vein or in the subclavian vein [7]. Thrombosis complications due to central venous catheter placement are categorized into three types: pericatheter thrombus , thrombotic occlusion of the catheter lumen, and mural thrombosis, including deep vein thrombosis (DVT) [8]. Deep vein thrombosis happens when a thrombus (blood clot) forms in the deep vein, usually in the lower limb. The incidence of deep vein thrombosis occurs in 1% to 5% of patients with central venous catheters. Deep vein thrombosis usually manifests with swelling and pain in the lower legs [8,9].

## Patient and observation

**Patient information:** the patient, Mr. S was 50 years old, lived in Tambak Asri, was married, self-employed, with high school education, Javanese, Muslim. He had been treated at the Dr. Soetomo Hospital on November 21, 2018. The patient presented to the hospital complaining of a swelling on the right leg which occurred 5 days before hospital admission. The swelling had become more prominent over time and was accompanied by pain in the right leg which occurred 2 days before admission. Pain affected the entire right leg, and was intermittent. In addition, the patient also complained of intermittent shortness of breath which occurred one month before. However, the patient did not have shortness of breath when he was admitted to the hospital. Fever, cough, weight loss, decreased appetite, and night sweats were not reported. The amount of urine produced in a day was 200-300 ml and no defecation problem was reported. The patient had a history of pulmonary tuberculosis (TB) successfully treated in 2015.

**Clinical findings:** the patient had a history of hospitalization at the Dr. Soetomo Hospital 1 week before due to chronic kidney disease (CKD) stage V + refractory hyperkalemia + left solitary kidney + left kidney stone + left ureteral stone + bladder stone + community acquired pneumonia (CAP) + stage II hypertension + obstruction syndrome post TB. He had undergone lumen catheter insertion in the right femoral vein (previous treatment) and removed when re-hospitalized. He underwent hemodialysis during the previous treatment (10^th^ November 2018). A family history of diabetes, hypertension, heart disease, liver, and kidney disease was not reported. During the previous treatment, the patient was scheduled for *vesicolithotripsy*+ ureteroscopy (URS), but the patient refused it.

On physical examination, he was compos mentis, blood pressure was 100/70 mmHg, pulse rate was 98 beats per minute, respiration rate 24 breaths per minute, body temperature 36.9°C, SpO_2_96% (free air), body mass index (BMI) 22.9 kg/m^2^ and Wong-Baker pain scale was 3. He had pebral conjunctiva (-/-), icterus (-), cyanosis (-), dyspnea (-); single S1S2, murmur (-), gallop (-); inspection (left lung), increased palpation in the right upper lung, dullness in the right upper lung, vesicular/vesicular breath sound, rhonchi (-/-), wheezing (+) in the right lower lung. There were soepel, normal bowel sounds on abdominal examination, liver and spleen were not palpable, shifting dullness (-), muscular defense (-). On examination of the right lower extremity, there was edema (+), redness (+), it was warm (+), and Wells' score was 3.

Supporting examination results showed that haemoglobin was 11.4 g/dl, erythrocytes were 4.01 x 10^6^/ul, leukocytes 27.99 x 10^3^/ul, platelets 317 x 10^3^/ul, BUN 60 mg/dl, serum creatinine was 9.93 mg/dl, albumin 3.27 gram/dl, random blood glucose 125 mg/dl, sodium 136 mmol/L, potassium 6 mmol/l, chloride 95 mmol/l, HbSAg rapid test is (-), serum glutamic-oxaloacetic transaminase (SGOT) 28 U/l, SGPT 34 U/l, PPT 10.0 seconds, activated activated partial thromboplastin time (APTT) 30.5 seconds. Urinalysis: glucose (-), ketones (-), bilirubin (-), SG 1.005, blood +4, pH 7, protein (-), urobilinogen (-), nitrite (-), leukocytes +3, erythrocytes 25-50/lp, leukocytes 15-20/hpf. Blood gas analysis: pH 7.32, pCO_2_45 mmHg, pO_2_67.2 mmHg, HCO_3_20 mmol/L, BEecf -4.4 mmol/L, oxygen saturation (SO_2_) 96%. Sputum examination on November 3, 2018 did not revealed any acid resistant bacteria, and electrocardiography (ECG) examination detected sinus tachycardia. Radiological examinations were based on: 1) Anteroposterior (AP) chest X-ray which showed upper lobe atelectasis and right lung fibrosis, pulmonary TB sequelae, right pleural effusion, and no abnormalities were seen; 2) AP/lateral pedis and cruris right position photo with no abnormal results; 3) AP and lateral radiographs of the right femur showed no abnormality, ultrasound objectified solitary kidney on the left, mild hydronephrosis and left hydroureter which could be caused by left renal pelvis stones, bladder stones; 4) multislice computed tomography (MSCT) stonography showed left solitary kidney, left renal pelvis stone and left medial 1/3 stone causing left 1/3 proximal hydroureter and left mild hydronephrosis, bladder stone.

### Timeline of current episode

**Second day of treatment:** complaints of swelling with intermittent right leg pain, shortness of breath (-). Physical examination: compost mentis, blood pressure 110/70 mmHg, pulse rate 98 beats per minute, respiration rate 24 breaths per minute, temperature 36.7°C, SpO_2_98% free air, urine production 650 ml/24 hours (drinking 600 ml + correction fluid 60 ml), lower extremity dextraedema (+), redness (+), and warm (+). Laboratory tests: BUN 52 mg/dl, serum creatinine 7.01 mg/dl, sodium 132 mmol/l, potassium 6.8 mmol/l, chloride 91 mmol/l. Assessment: acute on CKD + sepsis et cause urinary tract infection (UTI) + suspected DVT in the right lower extremity associated with differential leucocyte count (DLC) + hyperkalemia (6.8 mmol/l) + left mild hydronephrosis with medial 1/3 left hydroureter et causa left renal stone and ureteral stone + bladder stones + left solitary kidney + controlled hypertension + pulmonary tuberculosis sequelae. Therapy planning: three-step treatment of hyperkalemia (ca gluconate 1 ampoule IV injection followed by D40 + 2 units of insulin as part every 30 minutes for 3 times), nebulization of salbutamol 2.5 mg + ipratropium bromide every 8 hours, fluid restriction (maximum drink of 600 ml), other therapy remains. Planning diagnosis: complete blood count (CBC) evaluation, potassium serum post-correction (5.2 mmol/l), serial renal function test (RFT), and D-dimer. Monitoring included: clinical examination, vital signs, and urine production.

**Third-day of treatment:** complaints of swelling with intermittent right leg pain, shortness of breath (-). Physical examination: compost mentis, blood pressure 110/70 mmHg, pulse rate 93 beats per minute, respiration rate 20 breaths per minute, temperature 36.7°C, SpO_2_98% free air, urine production 1000 ml/24 hours (drinking 600 ml + correction fluid 85 ml), right lower extremity edema (+), redness (+), and warm (+). Laboratory tests: BUN 50 mg/dl, serum creatinine 6.89 mg/dl, sodium 135 mmol/l, potassium 6.2 mmol/l, and chloride 87 mmol/l. Assessment: acute on CKD + sepsis et causa UTI + suspected DVT in the right lower extremity associated with DLC + hyperkalemia (6.2 mmol/l) + left mild hydronephrosis with medial 1/3 left hydroureter and cause left renal stone and ureteral stone + bladder stones + left solitary kidney + controlled hypertension + pulmonary tuberculosis sequelae. Therapy planning: two-step treatment of hyperkalemia (ca gluconate 1 ampoule iv injection followed by D40 + 2 units of insulin as part every 30 minutes for 2 times), IVFD D5 + 10 units of insulin as part 7 drops per minute, fluid restriction (drinking a maximum of 1000 ml), other therapies. Planning diagnosis: CBC evaluation, potassium serum post correction (5.0 mmol/l), serial RFT, and D-dimer. Monitoring: clinical examination, vital signs, and urine production.

**Fourth-day of treatment:** complaints: swelling accompanied by intermittent pain in the right leg, shortness of breath (-). Physical examination: compost mentis, blood pressure 120/90 mmHg, pulse rate 96 beats per minute, respiration rate 20 breaths per minute, temperature 36.1°C, SpO_2_98% free air, urine production 1400 ml/24 hours (drink 1000 ml + infusion 500 ml + 60 ml correction fluid), lower extremity dextraedema (+), redness (+), and warm (+). Laboratory tests showed hemoglobin 10.4 g/dl, erythrocytes 4.12 x 10^6^/ul, leukocytes 23.25 x 10^3^/ul, platelets 378 x10^3^/ul, BUN 44 mg/dl, serum creatinine 6.51 mg/dl, sodium 135 mmol/l, potassium 4.8 mmol/l, chloride 95 mmol/l, D-dimer >4 g/ml (normal <0.5 µg/ml). Assessment: acute on CKD + sepsis et cause UTI + suspected DVT right lower extremity associated with DLC + corrected hyperkalemia (4.8) + left mild hydronephrosis with medial 1/3 left hydroureter and cause left renal stone and ureteral stone + bladder stone + left solitary kidney + controlled hypertension + pulmonary tuberculosis sequelae.

**Diagnosis:** complete blood count, serial RFT, Doppler ultrasound of the right lower extremity (scheduled in 28^th^ November 2018), and vascular duplex ultrasound scanning (DUS) echo (scheduled in 30^th^ November 2018). Therapy planning and monitoring were the same.

**Sixth-day of treatment:** complaints of swelling accompanied by intermittent pain in the right leg, and shortness of breath (-). On physical examination, the patient was compost mentis, blood pressure 130/90 mmHg, pulse rate 98 beats per minute, respiration rate 20 breaths per minute, temperature 36.2°C, SpO_2_97% free air, urine production 1500 ml/24 hours (infusion 500 ml + 1000 ml drink), lower extremity dextraedema (+), redness (+), and warm (+). Assessment, diagnosis, therapy, and monitoring were the same.

**Seventh-day of treatment:** complaints of swelling accompanied by intermittent pain in the right leg. Physical examination, the patient was compost mentis, blood pressure 130/90 mmHg, pulse rate 97 beats per minute, respiration rate 20 breaths per minute, temperature 36.7°C, SpO_2_99% free air, urine production 2000 ml/24 hours (infusion 500 ml + 1500 ml drink), right lower extremity edema (+), redness (+), and warm (+). Laboratory tests showed hemoglobin 11.3 g/dl, erythrocytes 4.48 x 10^6^/ul, leukocytes 15.38 x 10^3^/ul, platelets 375 x 10^3^/ul, BUN 20 mg/dl, serum creatinine 1.52 mg/dl, sodium 137 mmol/l, potassium 4.8 mmol/l, and chloride 91 mmol/l. Assessment: acute on CKD + sepsis et cause UTI + suspected DVT in the right lower extremity associated with DLC + corrected hyperkalemia (4.8) + left mild hydronephrosis with medial 1/3 left hydroureter and cause left renal stone and ureteral stone + bladder stone + left solitary kidney + controlled hypertension + pulmonary tuberculosis sequelae. Diagnosis, therapy, and monitoring were the same.

**Eigth-day of treatment:** complaints of swelling accompanied by intermittent pain in the right leg. On physical examination, the patient was compost mentis, blood pressure 130/80 mmHg, pulse rate 89 beats per minute, respiration rate 20 breaths per minute, temperature 37.0°C, SpO_2_99% free air, urine production 2000 ml/24 hours (infusion 500 ml + 1500 ml drink), right lower extremity edema (+), redness (+), and warm (+). Assessment: acute on CKD + sepsis et causa UTI + DVT in the right lower limb associated with DLC + corrected hyperkalemia (4.8) + left mild hydronephrosis with medial 1/3 left hydroureter and cause left renal stone and ureteral stone + bladder stone + left solitary kidney + controlled hypertension + pulmonary tuberculosis sequelae. Diagnosis: Doppler ultrasound of right lower extremity (today), while others were the same. Therapy: venflon, other therapies were same. Monitoring was constant.

**Ninth-day of treatment:** complaints of swelling accompanied by intermittent pain in the right leg. On physical examination, the patient was compost mentis, blood pressure 130/80 mmHg, pulse rate 89 beats per minute, respiration rate 20 breaths per minute, temperature 36.7°C, SpO_2_98% free air, urine production 1500 ml/24 hours (drinking of 1500 ml), lower extremity dextraedema (+), redness (+), and warm (+). Patient assessment: acute on CKD + sepsis and cause UTI + suspected deep vein thrombosis (DVT) in the right lower extremity associated with DLC + corrected hyperkalemia (4.8) + left mild hydronephrosis with medial 1/3 left hydroureter and cause left renal stone and ureteral stone + bladder stone + left solitary kidney + controlled hypertension + pulmonary tuberculosis sequelae. Diagnosis: waiting for result of Doppler ultrasound of the right lower extremity. Therapy and monitoring were the same.

**Tenth-day of treatment:** complaints of swelling accompanied by intermittent pain in the right leg. Physical examination: compost mentis, blood pressure 130/80 mmHg, pulse rate 89 beats per minute, respiration rate 20 breaths per minute, temperature 36.8°C, SpO_2_98% free air, urine production 1200 ml/24 hours (drinking 1200 ml), lower extremity dextraedema (+), redness (+), and warm (+). Laboratory tests showed hemoglobin 10.3 g/dl, hematocrit 32.8%, erythrocytes 3.65 x 10^6^/ul, leukocytes 10.23 x 10^3^/ul, platelets 168 x 10^3^/ul, BUN 12 mg/ul. Dl, serum creatinine 0.89 mg/dl, sodium 133 mmol/l, potassium 3.6 mmol/l, chloride 99 mmol/l. Patient assessment: acute on CKD + post sepsis + suspected deep vein thrombosis (DVT) in the right lower extremity associated with DLC + left mild hydronephrosis with medial 1/3 left hydroureter and cause left renal stone and ureteral stone + bladder stone + left solitary kidney + controlled hypertension + pulmonary tuberculosis sequelae. Planning diagnosis: waiting for the right lower extremity Doppler ultrasound results, vascular DUS echo (today), others tests were the same. Therapy planning and constant monitoring were the same.

**Thirteenth day of treatment:** complaints of swelling accompanied by intermittent pain in the right leg. Physical examination: compost mentis, blood pressure 130/80 mmHg, pulse rate 90 beats per minute, respiration rate 20 breaths per minute, temperature 36.7°C, urine production 1200 ml/24 hours (drinking 1200 ml/24 hours), lower extremities dextraedema (+), redness (+), and warm (+). Doppler ultrasound of the right lower extremity showed: 1) Diffuse deep vein thrombosis from the common femoral vein, great saphenous vein (GSV), common femoral, superficial, popliteal vein, into right distal posterior tibial vein with subcutaneous edema at the level of the femoral artery to the right dorsalis pedis; 2) no stenosis/occlusion/vascular malformation of the arterial system was seen. Vascular ultrasound (DUS) showed deep vein thrombosis in the right lower extremity. Laboratory results: INR 0.93. Assessment: acute on CKD + post sepsis + deep vein thrombosis (DVT) in the right lower extremity associated with DLC + left mild hydronephrosis with medial 1/3 left hydroureter and cause left renal stone and ureteral stone + bladder stone + left solitary kidney + controlled hypertension + pulmonary tuberculosis sequelae. Planning diagnosis: INR evaluation. Treatment: fondaparinux injection 7.5 mg every 24 hours im (first day).

**Fourteenth day of treatment:** complaints: swelling accompanied by intermittent pain in the right leg. Physical examination showed compos mentis, blood pressure 130/80 mmHg, pulse rate 92 beats per minute, respiration rate 20 breaths per minute, temperature 36.0°C, urine production 1200 ml/24 hours (1200 ml drinking), lower extremity dextraedema (+), redness (+), and warm (+). The assessment was the same. Diagnosis: INR evaluation. Therapy: fondaparinux 7.5 mg injection for 24 hours (second day), the other therapy was the same.

**Fifteenth day of treatment:** complaints: reduced swelling with pain in the right leg. Physical examination showed compos mentis, blood pressure 130/80 mmHg, pulse rate 90 beats per minute, respiration rate 20 breaths per minute, temperature 36.7°C, urine production 1200 ml/24 hours (1200 ml drinking), reduced lower extremity dextraedema. The assessment was the same. Diagnosis: INR evaluation. Therapy: fondaparinux 7.5 mg injection in 24 hours im (third day), warfarin 2 mg/24 hours (first day), the other therapy was the same.

**Sixteenth day of treatment:** complaints: reduced swelling and pain in the right leg. Physical examination showed compos mentis, blood pressure 130/80 mmHg, pulse rate 90 beats per minute, respiration rate 20 breaths per minute, temperature 37.2°C, urine production 1500 ml/24 hours (drinking 1500 ml), reduced dextra edema in lower extremities. Laboratory test results: INR 1. Diagnosis: INR evaluation. Therapy: fondaparinux injection 7.5 mg/24 hours im (fourth day), warfarin 2 mg/24 hours (second day), other therapy.

**Eighteenth day of treatment:** complaints: swelling and pain in the right leg is reduced. Physical examination showed compos mentis, blood pressure 130/80 mmHg, pulse rate 92 beats per minute, respiration rate 20 breaths per minute, temperature 37.0°C, reduced dextra lower extremity edema. Laboratory: INR evaluation. Patient assessments were the same. Diagnosis: RFT evaluation, INR evaluation. Treatment: fondaparinux injection 7.5 mg/24 hours im (sixth day), warfarin 2 mg/24 hours (fourth day), other therapy.

**Twenty-first day of treatment:** complaints: swelling and pain in the right leg is reduced. Physical examination showed compos mentis, blood pressure 130/80 mmHg, and rate of 92 beats per minute, respiration rate of 20 breaths per minute, temperature 37.0°C, reduced dextraedema in lower extremities. Laboratory: INR 2.5. The patient assessment was the same. Diagnosis: none. Therapy: injection of fondaparinux 7.5 mg/24 hours im (stop), warfarin 2 mg/24 hours (seventh day), other therapy.

**Diagnostic assessment:** assessment: atelectasis, right pulmonary fibrosis, TB sequelae and right pleural effusion. There were no signs of active pulmonary tuberculosis on clinical, radiological, and microscopic assessment. Diagnosis: spirometry when the patient was stable. Treatment planning: O_2_nasal cannula 3-4 liters per minute (lpm) prn, nebulized salbutamol 2.5 mg + ipratropium bromide every 8 hours. Thoracic-cardiovascular surgery consultation: Doppler ultrasound of the right lower extremity and elastic bandage. Heart consultation: DVT in the right lower extremity has not been ruled out. Diagnosis: echo vascular duplex ultrasound (DUS). Treatment: right lower extremity compression splinting.

**Diagnosis:** initial assessment showed acute CKD + sepsis et cause UTI + suspected deep vein thrombosis (DVT) in the right lower limb associated with DLC + hyperkalemia (6.0 mmol/l) + left mild hydronephrosis with left medial 1/3 hydroureter and cause left renal stone and ureteral stone + bladder stones + left solitary kidneys + controlled hypertension + pulmonary tuberculosis sequelae. Initial therapy planning included high-calorie diet (2100 calories per day) low in protein and salt, no fruit, vegetables, broth; venflon/syringe only, 2-step treatment of hyperkalemia (ca gluconate 1 ampoule iv injection, followed by D40 + 2 units of insulin as part twice every 30 minutes), injection of ceftriaxone 1 gram every 12 hours iv, injection of ranitidine 50 mg every 24 hours iv, paracetamol 500 mg every 8 hours prn, amlodipine 10 mg every 24 hours, and fluid restriction (drink maximum 600 ml/24 hours). Diagnosis was based on complete blood count, repeated RFT l, post-corrected serum potassium (5.3 mmol/l), blood culture, urine culture, and D-dimer. Monitoring was based on clinical examination, vital signs, and urine production.

**Informed consent:** the informed consent is provided by the Dr. Soetomo Hospital and given to the patient. The patient agreed to the informed consent on November 21, 2018 at 12.00 pm for examination in Pandan I Room, Class III.

## Discussion

Vascular access is an essential part of hemodialysis and consists of three steps; arteriovenous fistulas, synthetic grafts, and DLC installation. The use of arteriovenous fistula and synthetic graft is recommended for permanent vascular access. Differential leucocyte count (DLC) is often used, especially in patients who are on dialysis for the first time, to replace a temporary catheter or a blocked DLC, arteriovenous fistula failure, and for temporary access [5]. Differential leucocyte count (DLC) placement was performed by placing a catheter in the central vein in the internal jugular vein, femoral vein or subclavian vein. Femoral catheter placement is an option when urgent hemodialysis or temporary access are needed because femoral vein catheter placement is much easier than subclavian or internal jugular catheter placement [10,11].

Complications in the installation of the central venous access can be classified into immediate and late complications. Immediate complications include: 1) Hematoma around the insertion site; 2) bleeding; 3) embolism; 4) cardiac dysrhythmias. While further complications that can occur are: 1) infection (central line-associated bloodstream infection/CLABSI); 2) phlebitis; 3) deep vein thrombosis; and 4) venous perforation [11]. The patient underwent DLC insertion in the femoral vein as first access to hemodialysis. The patient had an advanced complication called deep vein thrombosis that occurred one week after femoral access installation. Thrombosis associated with the use of central venous catheter is classified into: 1) Thrombosis of pericatheter sheath; 2) thrombosis of catheter lumen and; 3) mural thrombosis, including deep vein thrombosis. Deep vein thrombosis occurs when a blood clot known as a thrombus forms in a deep vein [8,12].

The pathophysiology of catheter-related thrombosis begins with central venous catheter insertion that causes local damage to the vein at the access site. Few hours after the insertion, fibrin deposition on the surface of the catheter, as a thrombogenic factor, is found. The deposition consists of blood clots and protein that occur along the catheter sheath at insertion or venotomy. As a result, there is a reduction in blood flow of up to 60% around the central venous catheter leading to cellular adhesion to the catheter and vein walls. Ultimately, endothelial erosion triggers the formation of a mural thrombus, which covers the vein lumen over time [8,13].

The risk factors for deep vein thrombosis are divided into catheter-related and patient-related factors. Catheter-related risk factors are: 1) Peripherally inserted central catheter (PICC); 2) femoral access; 3) larger catheter diameter; 4) multi-lumen, and; 5) location of the catheter tip proximal to the superior vena cava. While the risk factors associated with the patient are: 1) Patient with age >60 years; 2) BMI >25 kg/m^2^; 3) presence of comorbid cancer, especially metastases; 4) chemotherapy; 5) thoracic radiotherapy; 6) previous central catheter-related thrombosis; 7) previous central venous catheter insertion; 8) severe disease; 9) systemic or catheter-related infections, and; 10) acquired hypercoagulability. In addition, several other risks factors may be associated. However, the results are inconsistent with studies, whether significant or not, such as: 1) diabetes; 2) hypertension; 3) smoking, and; 4) previous surgery history [14,15].

Catheter insertion in the femoral vein is a risk factor related to the use of a catheter. In contrast, a risk factor related to the patient is the presence of a systemic infection (in this case, sepsis). Both of these factors are known to be risk factors that increase the incidence of deep vein thrombosis. Patients also had comorbid hypertension, which increased the risk of deep vein thrombosis. A study stated that symptomatic thrombosis occurs in 1-5% of patients with central venous catheters, whereas Doppler ultrasound (DUS) or contrast venography revealed asymptomatic deep vein thrombosis in 41% and 19% of patients. Thus, deep vein thrombosis is a quite common complication in patients with central venous catheter [8]. The initial manifestation of deep vein thrombosis is that although medication and fluids flow into the catheter, blood cannot be drawn backwards. This happens because the catheter tip with a thrombus forms a one-way valve phenomenon that can inhibit the blood return from the catheter, while dialysis fluid flow is still possible. The more the thrombus gets bigger, the greater symptoms occur patients´ symptoms of deep vein thrombosis in legs are: 1) Pain; 2) swelling; 3) redness and warmth; 4) enlargement of superficial veins due to attempts to bypass the thrombus area, and; 5) pain from a simple touch [13].

Supporting investigation with duplex ultrasound was used for the diagnosis of deep vein thrombosis. However, prior suspicion of deep vein thrombosis, can be based on clinical criteria (using Wells' score): active cancer (receiving treatment in the last six months or receiving palliative therapy); paralysis, paresis, or immobilization of the lower extremities; three-days bed rest or surgery requiring regional or general anesthesia in the last 12 weeks; local pain along with the distribution of the deep venous system; swelling (whole leg swollen); calf swelling 3 cm bigger than the asymptomatic contralateral side (10 cm below the tibial tuberosity); unilateral pitting edema; superficial collateral veins; more alternatives other than DVT.

If there is a differential diagnosis similar to deep vein thrombosis, the score is -2. The interpretation of Wells' score results is divided into: 1) Low probability with a score of -2 to 0; 2) moderate probability with a score of 1 to 2, and; 3) high probability with a score of 3 to 8. A low Wells' score cannot rule out the possibility of deep vein thrombosis or the clinician's opinion to start anticoagulation therapy in hospitalized patients. When the probability is low, further examination with D-dimer is required, and ultrasound examination is recommended. When Wells' score is moderate to high, an ultrasound examination is recommended [16].

Ultrasound examination is carried out by pressing the probe on a deep vein. It will collapse in a deep vein when the vein is compressed due to its smooth and flexible structure, whereas in a thrombus-filled deep vein, it will not collapse. Ultrasound examination can also evaluate the venous flow. The chosen ultrasound used DUS with a sensitivity of 94% and a specificity of 96% [9,12]. The patient has edema and pain accompanied by redness of the right leg as a manifestation of deep vein thrombosis. This is reinforced by patient's Wells´ score 3 (local pain, swelling of the entire right leg, and pitting edema). Based on scoring interpretation, it can be concluded that the patient is most likely to have DVT, and further examination using ultrasound is needed to confirm patient's diagnosis. After doing vascular DUS echo, deep vein thrombosis in the right lower extremity is found.

The management of DVT is based on anticoagulants. The standard treatment of DVT is the initial administration of low molecular weight heparin (LMWH) or fondaparinux at therapeutic doses followed by oral anticoagulation. Parenteral anticoagulation should be given for at least 5-7 days or longer. Parenteral drug can be discontinued when the international normalized ratio (INR) is 2.0-3.00 [17]. The minimum duration of anticoagulation therapy is three months, but the optimal administration is unknown. In addition to taking a long time, the response to therapy is individual. The function of anticoagulation therapy is to stabilize the thrombus and prevent further thrombosis as well as its early and subsequent complications. Early complications of DVT are widespread thrombosis, pulmonary embolism (PE), significant bleeding due to anticoagulants, and death. Subsequent complications include recurrent thrombosis, post-thrombotic syndrome (PTS), and pulmonary hypertension due to chronic PE [9,12].

For six days, the patient was given an anticoagulant (subcutaneous fondaparinux injection) followed by warfarin (an anticoagulant tablet). The patient was given warfarin for three months. The patient's complaints of swelling and pain in the right leg began to reduce and eventually disappeared.

## Conclusion

The authors have reported the case of a patient with right lower extremity DVT, which is a further complication of DLC insertion. In this case, the diagnosis was made based on clinical criteria and ultrasound investigations. The management of this patient was based on the administration of an anticoagulant (fondaparinux followed by warfarin). The use of DLC associated with an increase in DVT risk in these patients is femoral access, systemic infection, and comorbid hypertension.
